# Optimization of Immunotherapy Strategies Based on Spatiotemporal Heterogeneity of Tumour‐Associated Tissue‐Resident Memory T Cells

**DOI:** 10.1111/imm.13924

**Published:** 2025-03-20

**Authors:** Yile Shang, Yinjun He, Xiang Zhang, Wenguang He, Hanju Hua, Feng Ye, Xile Zhou, Yandong Li, Weixiang Zhong, Guosheng Wu, Weiqin Jiang

**Affiliations:** ^1^ Department of Colorectal Surgery, The First Affiliated Hospital Zhejiang University School of Medicine Hangzhou China; ^2^ College of Medicine Zhejiang University Hangzhou China; ^3^ Department of Radiology, First Affiliated Hospital Zhejiang University School of Medicine Hangzhou China; ^4^ Department of Pathology, First Affiliated Hospital Zhejiang University School of Medicine Hangzhou China

**Keywords:** clinical prognosis, immune checkpoint blockade (ICB), spatiotemporal heterogeneity, tissue‐resident memory T cells (TRMs), tumour‐infiltrating lymphocytes (TILs)

## Abstract

Tissue‐resident memory T cells (TRMs) reside in peripheral tissues and provide rapid immune defence against local infection and tumours. Tumour‐associated TRMs share common tissue‐resident features and formation mechanisms, representing some unique subsets of tumour‐infiltrating lymphocytes (TILs). However, differences in the tumour microenvironment(TME) and tumour evolution stage result in TRMs exhibiting temporal and spatial heterogeneity of phenotype and function not only at different stages, before and after treatment, but also between tumours originating from different tissues, primary and metastatic cancer, and tumour and adjacent normal tissue. The infiltration of TRMs is often associated with immunotherapy response and favourable prognosis; however, due to different definitions, it has been shown that some subtypes of TRMs can also have a negative impact. Therefore, it is crucial to precisely characterise the TRM subpopulations that can influence the therapeutic efficacy and clinical prognosis of various solid tumours. Here, we review the spatiotemporal heterogeneity of tumour‐associated TRMs, as well as the differences in their impact on clinical outcomes. We also explore the relationship between TRMs and immune checkpoint blockade (ICB) and TIL therapy, providing insights into potential new targets and strategies for immunotherapy.

## Graphical abstract

The tumour microenvironment (TME) and tumour evolution stage lead to temporal and spatial heterogeneity in tumour‐resident macrophages (TRMs), affecting their phenotype and function across different stages and tumour types. While TRMs infiltration is often linked to positive immunotherapy responses and prognosis, some subtypes may negatively impact outcomes due to varying definitions. Therefore, accurately characterising TRMs subpopulations is essential for understanding their influence on therapeutic efficacy and clinical prognosis in solid tumours. This review examines the spatiotemporal heterogeneity of TRMs, their clinical implications, and their relationship with immune checkpoint blockade (ICB) and TIL therapy, highlighting potential immunotherapy targets and strategies.

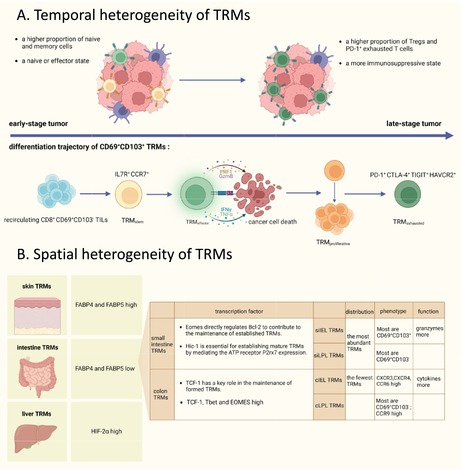

## 1 Introduction

Tissue‐resident memory T cells (TRMs) were first identified in 2009 as a special subgroup of memory T cells that do not enter the circulation but reside in the skin, providing immune protection against local infection [1]. Since then, TRMs have been found to exist widely in the skin, lung, reproductive tract, gastrointestinal tract, and other tissues, and have a strong immune protection against local inflammation or tumours [2, 3, 4]. In the tumour microenvironment(TME), TRMs can not only release perforin and granzyme to kill cancer cells directly, but also secrete cytokines such as IFN‐γ, IL‐2, and TNF‐α to promote the infiltration of other effective immune cells, such as dendritic cells (DCs), B cells, and natural killer cells (NK cells), which indirectly enhances the antitumour effect [5, 6]. However, due to the variations of TME, TRMs residing in it are highly spatiotemporal heterogeneous, which poses great challenges to its functional research and translational application.

Many studies have focused on the potential application of TRMs in tumour immunotherapy. Although the immune checkpoint blockade (ICB) therapy, represented by PD‐1/PD‐L1 antibody, has achieved success and significantly prolonged the survival of cancer patients, the response rate is low, only 20%–30%. The reason is that, in addition to PD‐1, a variety of checkpoints are involved in the immune escape of tumours. For example, with the increasing understanding of TRM functional subsets, researchers have found that lymphocyte activation gene 3(LAG‐3) and T‐cell immunoglobulin and mucin‐domain containing 3 (TIM‐3) are very important exhausted molecules, and new ICB strategies targeting these two markers have been successful and are on the way to clinical application. So, accurate identification of biomarkers for each functional subpopulation of TRMs can optimise target selection and provide more effective ICB strategies. Adoptive cell therapy (ACT), mainly represented by tumour‐infiltrating lymphocytes (TILs), is considered to have great potential in a variety of solid tumours, expected to open a new chapter in the field of cancer therapy. TILs are isolated from the tumour, then selected in vitro for amplification of tumour‐specific TILs, and ultimately infused intravenously back into the patients [7]. In this process, how to accurately select lymphocytes with anti‐tumour potential from the highly heterogeneous cell population obtained by isolation is the key to the success of TIL therapy. At present, the conventional method of selection is co‐culture with tumour cells, but the filter model guided by biomarkers is worth exploring to distinguish tumour‐specific TILs from CD8^+^ bystander TILs. In addition, TRMs play a significant role in predicting the clinical prognosis, either due to response to immunotherapy or because TRMs have a direct or indirect role in providing a robust immune defence or escape for tumours.

Herein, we will review the temporal and spatial heterogeneity of TRMs, focusing on their phenotype, function, and their potential role in tumour therapy and prognosis. We believe that an in‐depth study of the heterogeneity of TRMs is expected to predict prognosis more accurately, provide better strategies for ICB and TIL therapy, and realise accurate and personalised immunotherapy of tumours.

## 2 The Formation Mechanism of TRMs and Its Relationship With TILs

### 2.1 Identification Markers of TRMs

The identification of TRMs is based on the expression of specific transcriptomics and surface molecules, as well as the characteristics that reside in tissues. With the widespread use of single‐cell sequencing and spatial transcriptomes in cancer research, our understanding of these molecules of TMRs with various functional phenotypes in TME has reached a very precise dimension, some of which are TRM‐specific and some are shared with other T cell subsets such as central memory T cells (TCM) and effector memory T cells(TEM). However, residency in tissues and lack of migration ability are central features of the TRMs, and therefore, most studies still use the expression of CD69 and CD103, combined with the absence of cycling markers to define TRMs [8, 9].

CD69, which is upregulated upon T cell activation, plays a critical role in tissue retention by antagonising sphingosine 1‐phosphate receptor 1 (S1PR1) and blocking S1PR1‐mediated signals for export from tissues [10, 11, 12]. CD103, or integrin αE, is encoded by the *Itgae* gene and is expressed on multiple tumour‐associated CD8^+^ TIL subsets, which maintain TRMs in local tissues by binding to E‐cadherin on the surface of epithelial cells [13]. CD44 and CD49a are attached to the extracellular matrix and retain TRMs in the tissue [14].

The up‐regulation of chemokine receptors, such as CXCR3 and CXCR6, and transcription factors, such as Hobit (encoded by *Znf683*), Blimp1 (encoded by *Prdm1*) and Arylhydrocarbon receptor (Ahr) are also hallmark features of TRMs [15]. The down‐regulation of transcription factors, such as Kruppel‐like factor 2 (KLF2), Tbet, Eomes, and T‐cell factor 1 (TCF1), is also crucial during TRMs development and retention in tissues [15].

#### 2.1.1 Formation Mechanism of TRMs

Nowadays, most researches on TRMs focused on CD8^+^ tissue‐resident lymphocytes, whereas few have been done on CD4^+^ TRMs, and there are still gaps in the formation mechanisms for CD4^+^ TRMs.

Regarding the formation of CD8^+^ TRMs, studies have shown that they come from circulating naive CD8^+^ T cells or some characteristic precursor cells and have the ability to separate from tissues for recycling. Naive CD8^+^T cells are activated by exposure to antigen, differentiate into cytotoxic effector cells, and clonally expand; the effector cells migrate to the periphery to kill the target cells and then die, whereas a small fraction of them (5%–10%) remain and differentiate into different subpopulations of memory cells, including stem cell‐like memory T cells (TSCM), TCM, TEM, and TRM [8]. It has been found that CD8^+^CD103^+^ TRMs in the skin develop from early KLRG1^−^effector precursor cells that eventually fully mature within the epidermis, and these precursor cells migrate into the epidermis after entering the dermis in a chemokine‐dependent pattern during early effector responses, eventually expressing CD103 [16]. Intestinal TRMs also develop from KLRG1^−^CD127^+^ memory precursor effector cells [17]. The transcription repressor Bach2 mediates KLRG1^+^ effector CD8^+^ T cells losing KLRG1 and differentiating into KLRG1^−^ memory cells, which can migrate to the lung, liver, and the intraepithelial lymphocytes of the small intestine (siIEL) tissues, further differentiating into CX3CR1^−^tissue resident cells [18].

**FIGURE 1:**
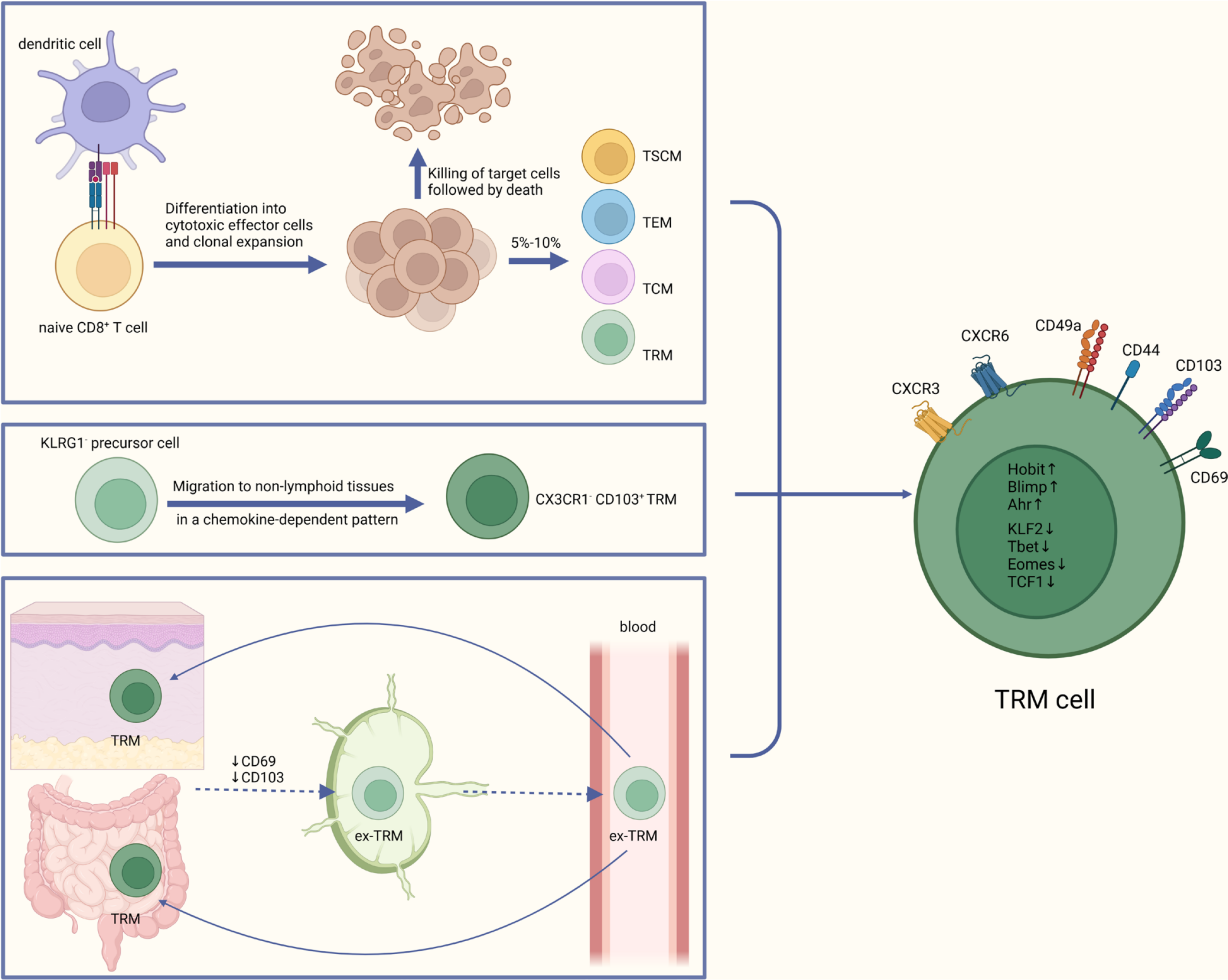
CD8+ TRMs originate from circulating naive CD8+ T cells or specific precursor cells and can detach from tissues for recycling.

Nevertheless, TRMs do not all permanently reside in specific tissues and can also return to circulation. TRMs are not at a stage of terminal differentiation but have developmental plasticity, and reactivation of established TRMs in the intestine can be achieved by down‐regulating CD69 and CD103, and then they recirculate as ex‐TRMs to generate TRM, TEM, and TCM cells while retaining the tendency to return to the primary tissue and regain the characteristics of TRMs [19, 20]. TRMs in the skin can be detached from the tissue and recirculate through the lymphatics [20]. Due to ex‐TRMs comprising less than 1% of the total peripheral blood mononuclear cells (PBMCs) in peripheral circulation, the ex‐TRMs detected in blood to date have primarily originated from skin and intestine tissues, which are rich in immune cells [21, 22]. The triggering factors and specific mechanisms of TRMs recirculation remain unclear, and this process can occur not only when tissues are in a stable state but also during inflammatory conditions, potentially due to antigen stimulation [22] (Figure 1).

**FIGURE 2:**
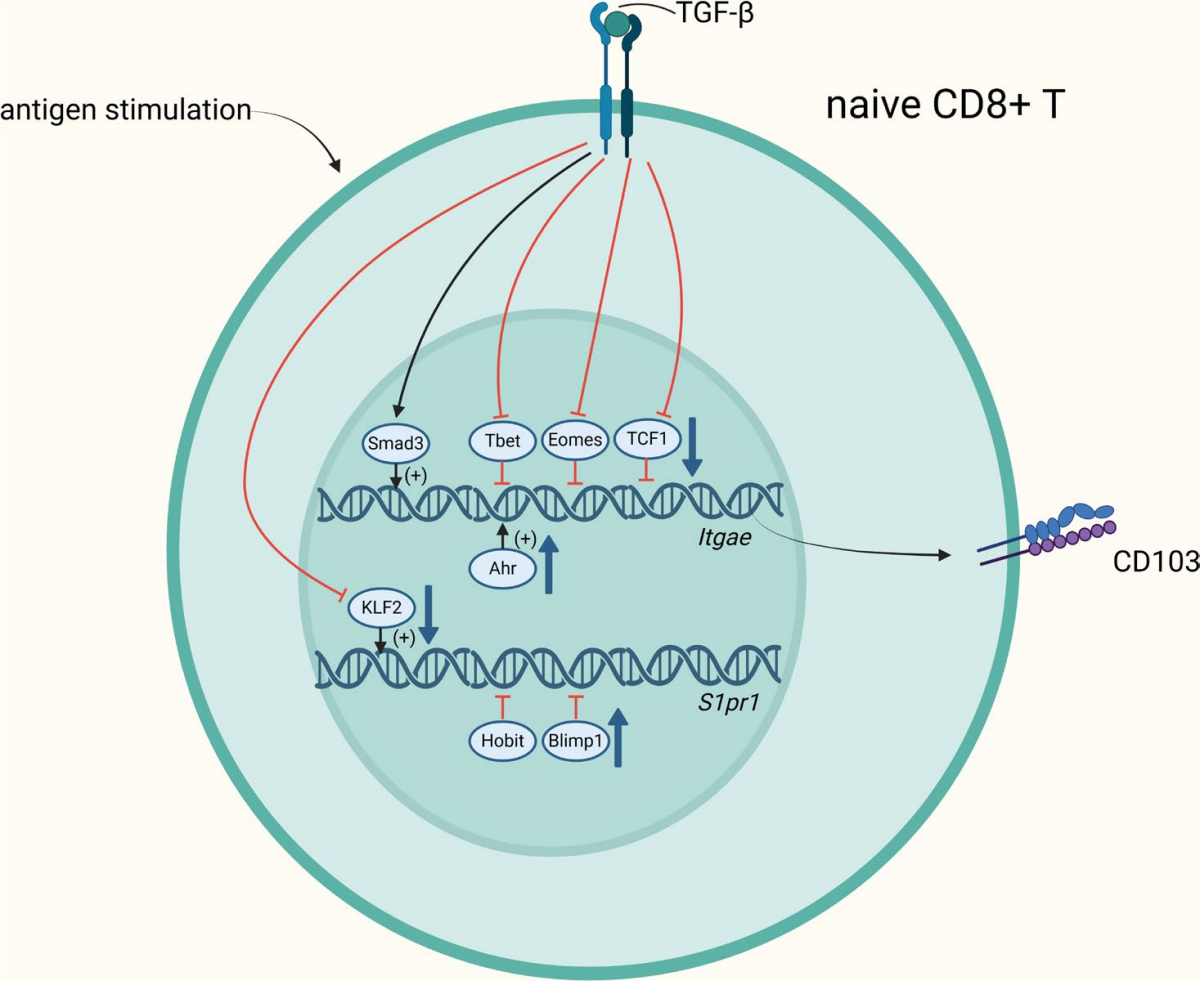
The characteristic transcription factors of TRMs bind to DNA, playing a crucial role in their formation and development.

The characteristic transcription factors of TRMs also participate in the formation and development of TRMs in a DNA‐binding manner [23, 24, 25]. Ahr directly binds to the gene loci of *Cd69* and *Itgae*, promoting the expression of TRM‐specific genes [23]. The transcription factors Hobit and Blimp1 extensively cooperate to bind to tissue egress genes such as *S1pr1* and *Klf2*, directly inhibiting the entry of CD8^+^ T cells into circulation [24]. Upon activation of naive CD8^+^ T cells during viral infection or antigen stimulation, transcription factors such as Hobit, Blimp1, and Ahr are upregulated, promoting differentiation toward TRMs [26]. Tbet, Eomes, and TCF1 directly bind to the *Itgae* gene loci in CD8^+^ T cells, inhibiting CD103 expression [25]. KLF2 directly binds to the promoter of the *S1pr1* gene and promotes its expression, which in turn facilitates the tissue egress of CD8^+^ T cells [27]. Integrin α_v_β_8_ expressed by DCs activates transforming growth factor‐β(TGF‐β) secreted by tumour cells in tissues and presents it to naive CD8^+^ T cells, and then Smad3 binds to the responsive element of the *Itgae* gene via the TGF‐β/Smad signalling pathway to directly induce CD103 expression [25]. This process can also inhibit the repressive functions of T‐bet, Eomes, and TCF‐1, indirectly promoting the expression of CD103 [25]. The down‐regulation of S1PR1, essential for the formation of TRMs, can be mediated by TGF‐β through the PI3K/Akt pathway, which subsequently downregulates the expression of KLF2 [10, 11, 12, 25] (Figure 2).

#### 2.1.2 Relationship Between TRMs and TILs

The first TIL therapy (Lifileucel) was approved for advanced melanoma on February 16, 2024, according to the results of the C‐144‐01 study, offering new hope for patients with solid tumour [28]. TILsare located in the TME and are mainly composed of CD8^+^ T cells, CD4^+^ T cells, B cells (including antigen‐presenting B cells, antibody‐producing B cells and regulatory B cells), NK cells, helper‐like innate lymphoid cells (ILCs) and so on [29]. Among them, CD8+ T cells have the most powerful anti‐tumour efficacy, which are also the main components of TRMs. Transcriptomic analysis of TILs in lung cancer shows that TRMs are enriched in tumours with a high density of TILs, and the expression of core signature genes in TILs is consistent with TRMs [30]. Therefore, TRMs are a distinct subpopulation of TILs. It has been noted that TRMs and TILs express similar tissue‐resident related genes, suggesting that TRMs may be predominant in TILs [7]. Similar to the heterogeneity of TRMs, TILs exhibit functional and quantitative diversity of infiltrating cells across different tumour types and even within the same lesion [31, 32].

The success of TIL therapy in solid tumours is due to its anti‐tumour potential and individualisation. The accurate selection of lymphocytes with anti‐tumour potential is crucial for TIL therapy. Currently, the traditional method of TIL selection is co‐culture with tumours. The resected tumour specimen, including tumour cells and lymphocytes, is divided into multiple fragments and cultured in cytokines, such as IL‐2, to stimulate T‐cell proliferation, resulting in eliminating tumour cells to form pure TIL cultures [33, 34]. This selection strategy retains the polyclonal nature and individualisation of TILs, as well as the anti‐tumour activity of TILs. However, this strategy cannot filter out bystander TILs, and may lose exhausted T cells that have potential anti‐tumour effects. Nowadays, many researchers have proposed that precise enrichment of TILs based on biomarkers, or combined with ICB strategy targeting the specific exhausted molecules to further improve the efficacy of TIL therapy. Thus, understanding the heterogeneity of TRMs can equally contribute to the optimisation of TIL therapy.

### 2.2 Temporal Heterogeneity of TRMs

#### 2.2.1 Variation of TRMs in Different Stages of Tumour and Differentiation Trajectories of TRMs

Analysis of primary and metastatic tissues from breast cancer patients revealed that the number of TILs enriched with large numbers of CD8^+^CD103^+^ TRMs was significantly higher in untreated primary breast cancer compared to metastatic tumour samples [35]. Similarly, a study of the relationship between the number of tumour‐infiltrating CD8^+^CD103^+^ TRMs and cancer progression in gastric cancer patients revealed that the number of TRMs in the early stages of tumour (TNM stage I, stage II) was significantly higher than in the advanced stages (TNM stage III and IV), suggesting that under natural conditions, the number of TILs gradually decreases as the tumour progresses [36]. Meanwhile, traditional treatments such as chemotherapy and radiotherapy may kill immune cells, weakening the anti‐cancer ability of TILs. Therefore, the efficacy of TIL therapy for advanced tumours after heavy treatment is currently unsatisfactory. Storing TILs isolated from surgical specimens of patients with naïve treatment as adjuvant therapy, or as first‐line treatment when the tumour recurs or metastasizes, would help to ameliorate this dilemma [37].

**FIGURE 3:**
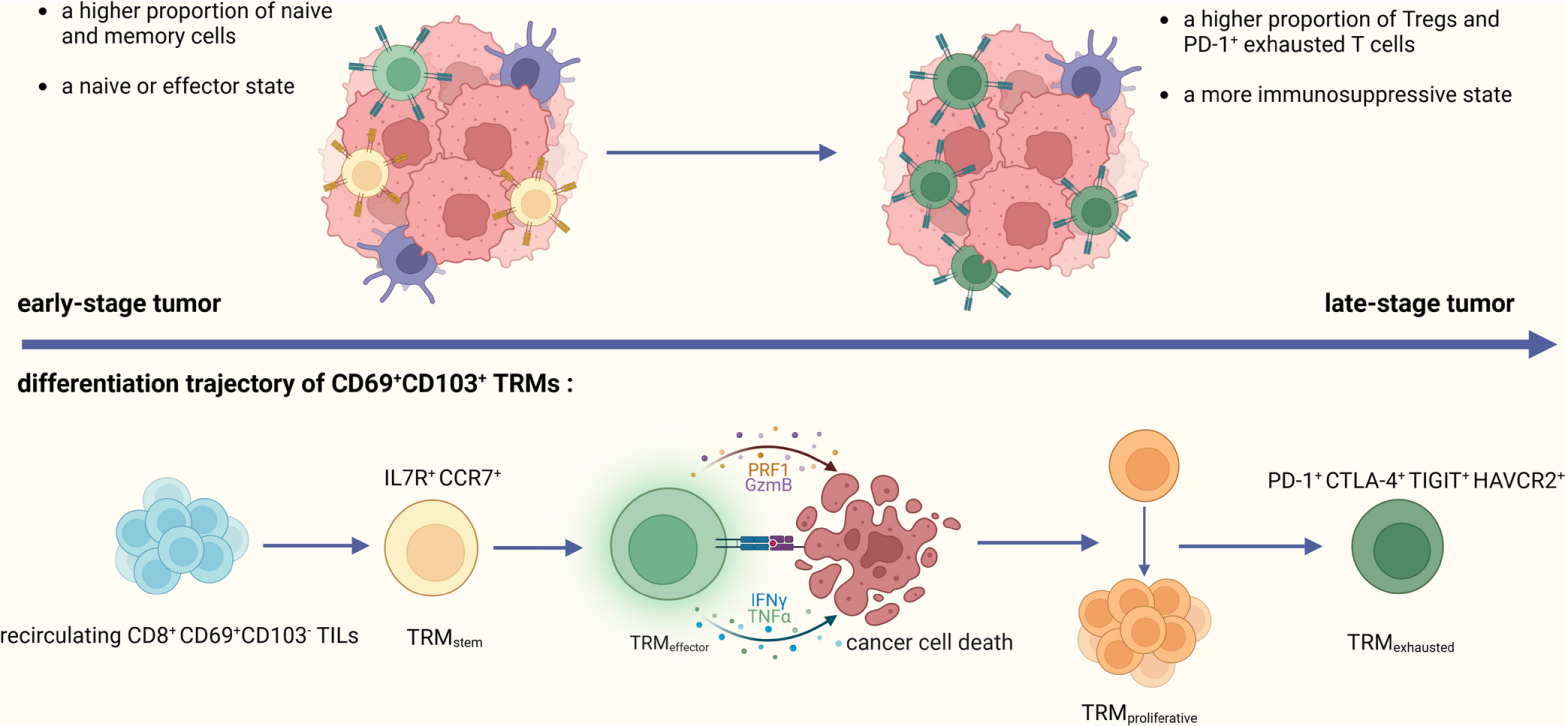
The transition of TILs from a naive or effector state in early tumours to an exhausted state in late tumours is consistent with the developmental stages of TRMs.

In late tumours, CD4^+^ and CD8^+^ TILs significantly reduced the proportion of naive and memory cells, as well as the expression of corresponding marker transcripts, while the proportion of exhausted CD8^+^ T cells increased, along with the upregulation of numerous co‐inhibitory receptor gene expression [38]. The transition of TILs from a naive or effector state in early tumours to an exhausted state in late tumours is consistent with the developmental stages of TRMs themselves. TRMs do not exist in a certain fixed effector or exhausted state within the tumour, but have a continuous process of transitional changes [39]. The differentiation and developmental trajectory of CD69^+^CD103^+^ TRM‐like cells can be summarised as follows: a small subset of recirculating CD8^+^ CD69^+^CD103^−^ TILs first differentiate into TRM stem cells with stem cell characteristics, which then differentiate into TRM effector cells capable of producing effector molecules such as GzmB, INF‐γ, TNF‐α, and subsequently further differentiate into TRM proliferative cells with highly proliferative features, ultimately becoming TRM exhausted [40]. The driver of this differentiation is thought to be the continuous antigenic and TCR stimulation that TRMs receive within the tumour tissues, ultimately resulting in their exhausted state during the late stages of cancer development [41]. Therefore, in late‐stage tumours, relieving the exhausted state of TRMs is anticipated to offer insights and rationale for the development of novel ICB therapeutic strategies (Figure 3).

Each stage of the differentiation pathway of TRMs is characterised by specific highly expressed markers. Genes associated with T cell stemness include chemokine receptor 7 (CCR7), transcription factor 7 (TCF7), interleukin‐7 receptor (IL7R), etc. Mediators associated with cytotoxicity include interferon (IFNG), granulysin (GNLY), granzyme B (GZMB), perforin 1 (PRF1), etc. Markers related to T cell proliferative potential include Inducible Co‐Stimulator (ICOS), Ki‐67, cyclin‐dependent kinase 1 (CDK1), etc. Molecules associated with exhausted TRMs may include programmed cell death 1 (PDCD1), cytotoxic T‐lymphocyte‐associated protein 4 (CTLA4), LAG‐3, V‐domain Ig suppressor of T cell activation (VISTA), B and T lymphocyte attenuator (BTLA), hepatitis A virus cellular receptor 2 (HAVCR2), Nuclear factor of activated T cells cytoplasmic1 (NFATC1), basic leucine zipper ATF‐like transcription factor (BATF), SLAM Family Member 7 (SLAMF7), SATB1, LY6E, CXCL10, CXCL13, etc. [42, 43].

#### 2.2.2 Variation of TRMs Before and After Therapy

Immunotherapy can enhance the anti‐tumour activity of CD69^+^CD103^+^ TRM‐like cells. In a variety of tumours, including breast and lung cancers, the number of CD69^+^CD103^+^ TRM‐like cells in tumours was significantly increased after ICB treatment targeting PD‐1 compared to pre‐treatment [44, 45, 46], enhanced production of interferon IFN‐γ and TNF‐α, with a significant increase in specific lysis of tumour cells [44]. It is evident that tumour‐specific TRMs are often in an inhibited state prior to immunotherapy, with different inhibitory or exhausted molecules. ICB targeting exhausted molecules can relieve the inhibited state of TRMs and make them strong cytotoxic potential. For example, the T cell Immunoreceptor with Ig and ITIM domains (TIGIT) is currently considered one of the most promising immune checkpoints, which binds its ligands CD155 and CD112 to form a complex regulatory network containing the TIGIT‐CD226 (a competing co‐stimulatory receptor delivering a positive signal) axis, initiating inhibitory signals and exerting immunosuppressive effects through multiple mechanisms [47, 48]. The analysis of TILs within the TME of endometrial cancer (EC), pancreatic ductal adenocarcinoma (PDAC) and urothelial bladder cancer (UBC) shows high infiltration of PD‐1^+^TIGIT^+^ TRMs, exhibiting features of terminally exhausted TILs [49, 50, 51]. In vitro and in vivo experiments, co‐blockade of PD‐1 and TIGIT reinvigorated the cytokine production capacity and cytotoxic efficacy of TRMs [49, 50, 52, 53].

Recently, due to the plasticity of the tumour immune microenvironment, an increasing number of studies have focused on its changes before and after radiotherapy [54]. Several preclinical studies have shown that radiotherapy can induce cancer cell death and promote tumour‐infiltrating DCs to recognise cancer cell‐derived DNA through the cGAS‐STING pathway, which in turn activates tumour‐specific T cells in tumour‐draining lymph nodes (TDLNs) [55]. This process recruits more CD8 + CD103+ TRMs to migrate to the tumour, converting it from a “cold” to a “hot” state, and transforming tumours that are unresponsive to anti‐CTLA‐4 into responsive states [55, 56]. Recently, several clinical studies have demonstrated that neoadjuvant chemoradiotherapy combined with immunotherapy can significantly enhance the complete response rate in patients with locally advanced rectal cancer [57, 58]. A clinical study also found that after radiotherapy, the CXCL13^+^CD8^+^ TRM subpopulation was activated and enriched, enhancing anti‐tumour effects and prolonging the overall survival (OS) and progression‐free survival (PFS) in patients with cervical cancer [59]. Traditionally, lymphocytes are considered the most sensitive cells to radiotherapy and will be killed quickly. However, radiotherapy can reprogram TILs in TME, increasing their radio‐resistance and allowing them to survive after radiotherapy [60]. Meanwhile, such radio‐resistant TILs exhibit enhanced spontaneous anti‐tumour characteristics, showing increased activity and an augmented ability to produce IFN [55, 60]. In summary, radiotherapy can not only directly eliminate tumour cells, but also has an activating effect on the immune system by promoting TILs infiltration, reprogramming TME, and enhancing the anti‐tumour activity of TRMs.

Currently, there is a lack of comprehensive descriptions regarding the changes in TRMs before and after chemotherapy and targeted therapy, indicating a need for further investigation. Preclinical and clinical studies on colorectal cancer have found that anti‐angiogenic treatment can significantly inhibit tumour cell proliferation in mice and enhance the infiltration of CD4^+^ and activated CD8^+^ T lymphocytes within tumours [61, 62]. Meanwhile, the increased TILs exhibited a significant rise in the expression of GZMB and GZME, along with a notable decrease in the expression of CX3C motif chemokine receptor 1 (*Cx3cr1*), indicating enhanced tumour‐killing activity [61, 62]. Another study using regorafenib for the treatment of triple‐negative breast cancer found that after treatment, there was not only an increase in CD4^+^ and CD8^+^ TILs within tumour tissues but also a trend toward increased CD8^+^ T cells and CD11b^+^CD11c^+^MHC II^high^ DCs in the spleen, suggesting that regorafenib may enhance the antigen‐presenting capacity of DCs [63]. In summary, chemotherapy and targeted therapy can modulate the immune microenvironment as systemic treatments by promoting the infiltration of TILs, enhancing their anti‐tumour immune responses, facilitating the presentation of tumour antigens, and so on.

### 2.3 Spatial Heterogeneity of TRMs

#### 2.3.1 Discrepancy Between Tumour and Adjacent Normal Tissues

CD8^+^CD103^+^ TRMs can be observed in tumour tissues, tumour‐adjacent tissues, and distant normal mucosa, and progressively increase with proximity to the tumour. In lung cancer patients, compared to homologous tumour tissues, adjacent normal tissues had fewer TRMs, lower expression levels of CD69, Ki67 (proliferation marker) and 4‐1BB (activation marker), suggesting that this population has not been recently exposed to specific antigens [45]. In patients with gastric cancer, it was likewise found that the expression of PD‐1 and 4‐1BB of TRMs in tumour tissues was significantly higher, whereas the expression of granzyme B and perforin was significantly lower than in non‐tumour tissues at least 5 cm away from the tumour, suggesting that TME of gastric cancer leads to variations of the phenotypes and cytotoxic function of TRMs [36]. The study of the distribution of TRMs in primary tumours, metastatic lymph nodes, peripheral blood, and uninvolved lymph nodes of patients with head and neck squamous cell carcinoma (HNSCC) revealed that high levels of CD8^+^CD39^+^CD103^+^ TRMs were present in both primary tumours and metastatic LNs. However, in peripheral blood and uninvolved LNs, CD8^+^CD39^+^CD103^+^ TRMs were almost absent [64]. In other studies of patients with cutaneous squamous cell carcinoma (cSCC) it has also been observed that the proportion of CD8^+^CD103^+^ T cells is increased in tumour tissues compared to non‐lesional skin and peripheral blood [65].

Single‐cell sequencing revealed that TRMs in both tumours and distant normal mucosa can be classified into two clusters, and the classification of TRMs is determined by the tissue environment. One is “active” TRMs, which have similar characteristics to TEMs, are functionally active, dominate in tumours, and are adapted to the inflammatory environment in TME to exert an immune effect [66]. The other is “quiescent” TRMs, which have similar characteristics to TCMs, are quiescent, long‐lived resident memory cells, with the expression of functional genes opposite to that of the active TRM, and dominate in the distal tumour‐free mucosa, which is conducive to long‐term retention and survival [66].

#### 2.3.2 Discrepancy in TRMs of Solid Tumours Across Tissue

TRMs rapidly adapt to their surrounding tissue microenvironment and exhibit different phenotypic and functional characteristics by specific alterations of surface molecules and transcriptional profiles, resulting in their discrepancy in solid tumours across tissue. Fatty acid binding proteins (FABP) can mediate exogenous free fatty acid (FFA) uptake to promote long‐term survival and anti‐infective function of TRMs [67]. The expression of FABP is tissue‐specific, that is, TRMs optimise the expression of FABP according to their tissue in which they reside, such as FABP4 and FABP5, which are highly expressed on the skin TRMs of mice, while poorly expressed on the intestine TRMs [67, 68]. Colonic TRMs are characterised by high expression of the transcription factors TCF‐1, EOMES, and Tbet, which is contrary to the classical TRMs transcription factor profile [15, 69, 70]. The transcription factors hypoxia inducible factor 1α(HIF‐1α)and HIF‐2α are essential for the survival and effector functions of T cells under low oxygen concentrations [71, 72]. The liver, as a unique immunogenic organ, contains hypoxic regions resulting from its distinct structure and blood supply system [73]. Therefore, in human liver sinusoids, due to hypoxia and TCR stimulation, CD69^+^ CD103^−^CD8^+^ TRMs, which represent the predominant population of CD8^+^ T cells, upregulate the expression of HIF‐2α, reflecting the tissue‐specific adaptability of TRMs [72].

In the intestine, TRMs exhibit heterogeneity across different intestinal tissues. An extensive comparative analysis of TRMs from four intestinal tissues, small intestinal epithelial & lamina propria (siIEL & siLPL) and colonic epithelial & lamina propria (cIEL & cLPL), revealed variations in TRMs distribution, phenotype and function: the most abundant TRMs were found in siIEL and siLPL, with the fewest in cIEL; the majority of siIEL CD8^+^ TRMs were CD69^+^CD103^+^ and exhibited high levels of both markers, while the majority of siLPL and cLPL TRMs expressed only CD69; TRMs in siIEL expressed higher levels of granzymes, whereas cIEL TRMs produced more cytokines [69, 70]. The transcription factor Eomes directly regulates Bcl‐2, which in turn significantly contributes to the maintenance of established small intestinal TRMs, while playing no significant role in the colon [69], where Eomes expression is higher. The transcription factor TCF‐1, on the other hand, has a key role in the maintenance of formed TRMs in the colon, but not in the small intestine [70]. Hic‐1 is predicted to be a gut‐associated transcription factor that is essential for the establishment of a mature TRMs population in the small intestine by mediating the expression of the ATP receptor P2rx7, but it may inhibit the formation of TRMs in other tissues such as salivary glands [74].

**FIGURE 4:**
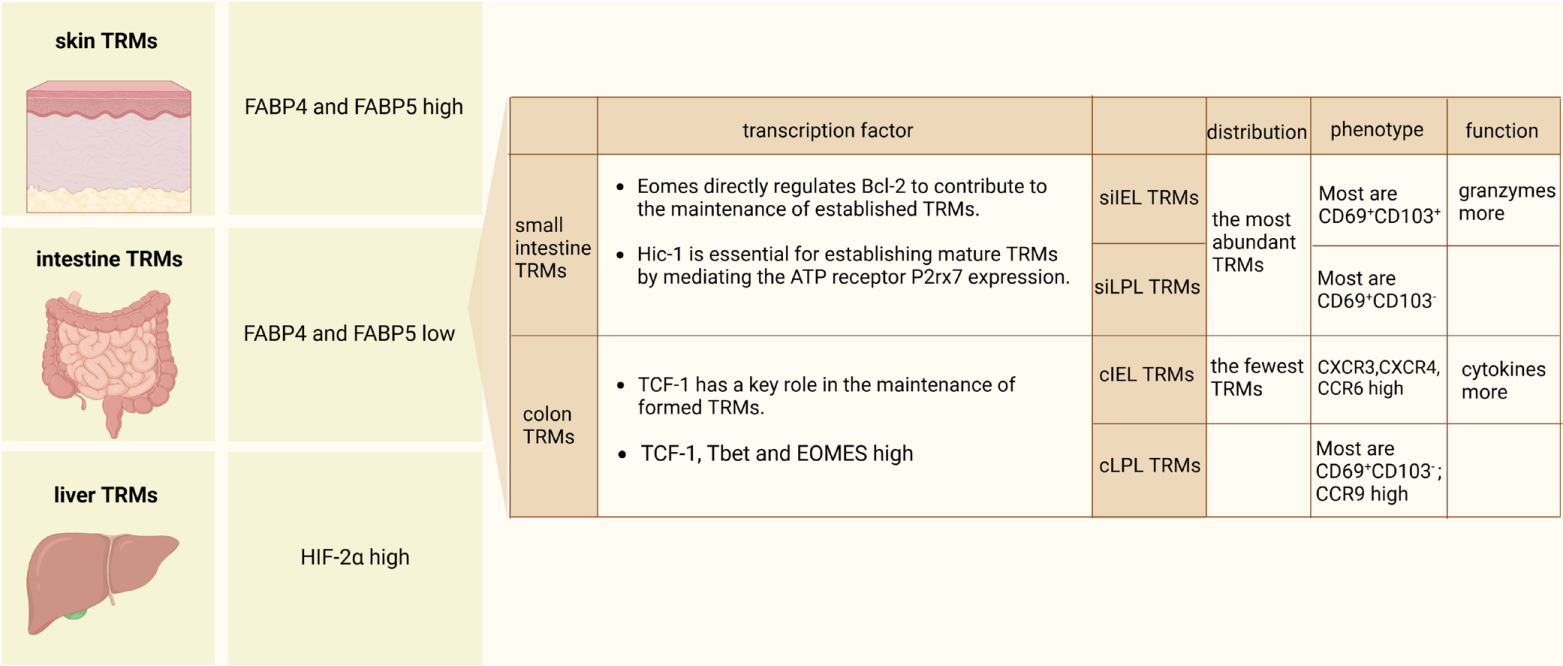
Discrepancy in TRMs of solid tumours across tissue.

TRMs from different organs or tissues are different; even from different anatomical regions of the same organ, they show heterogeneity in quantity, functional phenotype, and developmental plasticity. In intestinal tissues, TRMs from IEL produced higher levels of IFNγ than those from LPL, and cIEL produced the most IFNγ. In colonic TRMs, cIEL TRMs expressed CXCR3, CXCR4, CCR6, and Eomes to a greater extent and in a higher proportion, whereas cLPL TRMs expressed CCR9 in a higher proportion [69]. Meanwhile, siIEL TRMs have a higher plasticity to generate secondary TRMs than siLPL, which will express the phenotypic characteristics of the primary TRMs [69] (Figure 4).

### 2.4 Impact of TRMs on Clinical Prognosis

As TRMs not only specifically kill tumour cells but also provide long‐term immune defence, it is generally believed that patients with a high density of CD8^+^CD103^+^TRMs in their tumours are more responsive to immunotherapy, with a higher objective response rate (ORR) and a significantly increased progression‐free survival (PFS) [45, 46]. Several studies of triple‐negative breast cancer (TNBC) patients have indicated that genetic characterisation of CD8^+^CD103^+^ TRMs is associated with prolonged OS and reduced the risk of recurrence, and that breast cancer patients with higher levels of TRMs also have higher rates of pathologic complete response(pCR) toanti‐PD‐1 treatment [75]. In addition, similar conclusions can also be observed in a variety of solid tumours, such as head and neck squamous cell carcinoma (HNSCC) [76], oral squamous cell carcinoma (OSCC) [77], cervical cancer [78], lung cancer [79] and so on, confirming the potential role of TRMs for better prognosis due to a higher response rate to immunotherapy.

Nevertheless, there have been contradictory findings as to whether the presence of CD39^+^ TRMs contributes to clinical benefit in cancer patients. In TILs of cutaneous squamous cell carcinoma (cSCC), an increase in the proportion of CD103^+^ TRMs and the expression of CD103 is significantly correlated with the decrease in the time required for metastasis, which means that CD103^+^ TRMs in cSCC are associated with the progression of metastasis [65]. Comparing non‐small cell lung cancer (NSCLC) patients who were never‐smokers (NSs) with those who were ever‐smokers (ESs), tumours in the ESs group had a higher abundance and activation of CD45RO^+^ CD69^+^CD103^+/−^TRMs, yet tumours in this group were insensitive to combined ICB therapy and had a higher incidence of immune evasion events, leading to a worse prognosis [80]. The comparison between these studies suggests that the role of TILs in solid tumours may be more complex than traditionally conceived, and for different types of tumours, TRMs can exhibit either promoting or inhibiting effects on tumour progression. To explore the potential mechanism of TRMs leading to poor prognosis, it can be hypothesised that it may be due to the elevated level of immune selection pressure caused by TRMs, which induces tumours to evade the surveillance of the immune system through immune editing and eventually achieve tumour immune evolution and the occurrence of metastasis [81]. In preclinical models of NSCLC, high levels of pre‐existing TRMs showed a significant loss of MHC class I protein expression on tumour cells, and a greater proportion of tumour cells lost neoantigens, both of which are immune escape phenomena [80, 82]. It is evident that the pre‐existing TRMs prior to tumourigenesis drive immune escape, leading to their reduced response to ICB immunotherapy.

The effect of TRMs on the prognosis of solid tumours is controversial, possibly because the current definition and classification of TRMs are not accurate enough to reflect their heterogeneity. There are different functional subgroups of TRM in TME, and the dominant subsets vary across tumours, so their effects on tumour prognosis are bound to be different.

### 2.5 Effect of TRMs Heterogeneity on ICB and TIL Therapy

Up to now, CTLA‐4, PD‐1, and PD‐L1 antibodies have been widely applied in clinic, but the efficacy is still not satisfactory [83, 84]. In addition to these routine immune checkpoints, TRMs also highly express a variety of novel inhibitory molecules, which may become a new target for ICB therapy, among which LAG‐3, TIGIT, and TIM‐3 have been well studied. LAG‐3 is considered to be the most promising therapeutic target after PD‐1, and in March 2022, the US FDA approved the combination of LAG‐3 antibody Relatlimab and PD‐1 antibody Nivolumab for the treatment of unresectable or metastatic melanoma, announcing the arrival of an entirely new immune checkpoint inhibitor in nearly a decade [85, 86]. TIGIT inhibitor combined with PD‐1 antibody also demonstrated preliminary anti‐tumour activity in advanced solid tumours [48, 87]. As a checkpoint receptor, TIM‐3 interacts with its four ligands, inducing CD8+ T cell death and exhaustion through various mechanisms to reduce anti‐tumour immunity, and the efficacy of combining TIM‐3 and PD‐1 antibodies in different types of cancer is under evaluation, showing the advantage in improving antitumor immunity in both preclinical cancer models and clinical trials [88, 89, 90].

As well as the aforementioned molecules, there are many checkpoint molecules abundantly expressed on CD8+ TILs that may serve as potential targets for ICB therapy, such as NKG2A/HLA‐E, killer cell immunoglobulin‐like receptor 3DL3(KIR3DL3)/HHLA2, B and T lymphocyte attenuator (BTLA), V‐domain Ig suppressor of T cell activation (VISTA), ENTPD1(CD39), PVRIG, CD96, CXCL10, CXCL13and so on [83, 84]. In preclinical mouse model experiments, combined blocking of VISTA and PD‐1 can significantly prompt tumour regression, indicating its clinical value [91, 92]. Currently, novel checkpoints NKG2A and KIR3DL3 have been reported, and blocking their inhibitory activity is expected to provide new avenues for immunotherapy, and several clinical trials are recruiting patients for testing and are expected to generate preliminary promising results [93, 94].

In summary, the precise definition of TRMs functional subsets based on expressed molecules provides additional potential effective target options for the design of ICB therapy. This also reflects the importance of personalised precision immunotherapy based on precisely defined functional subgroups of TRMs.

In TIL therapy, TILs with anti‐tumour potential selected by co‐culture with tumor cells still need to be further filtered. Molecular markers of TRMs such as CD39 are widely used to filter out tumour‐specific TILs from CD8+ bystander TILs [29, 64, 95]. CD39(encoded by the ENTPD1 gene) is not only a marker of tumour‐specific TRMs; it has also been identified as a marker of activated or exhausted CD8^+^ TILs [96, 97]. Although CD39 + CD103+ TILs, with lower expression of genes involved in T‐cell recirculation, are in a state of antigen activation and clonal expansion, they have an increased expression of genes associated with T‐cell exhaustion, resulting in an exhausted phenotype [64, 98]. TIL therapy has now demonstrated good efficacy in the treatment of some solid tumours; however, the response rate and beneficiaries are still limited. Considering the heterogeneity of TRMs due to individual and tissue differences, intensive exploration of more novel biomarkers besides CD39 to accurately filter out tumour‐specific TILs will be more conducive to individualised immunotherapy. The transcription factor TCF‐1 inhibits the expression of effector substances and the cytotoxic potential of TRMs in the colon, so selecting TCF‐1 negative TILs for amplification has clinical significance in the inhibition of tumour growth and prolongation of disease‐free survival in colorectal cancer patients [70]. The transcription factor Runx3 is a key regulator necessary for the establishment of TRMs, and it has been found that after the adoptive transfer of the TILs that overexpress Runx3 into melanoma mouse models, the abundance of TRMs was increased, tumour growth was delayed, and the survival time of mice was prolonged [99]. For TIL therapy, investigating the molecular characteristics of tumour‐specific TRMs in different spatiotemporal conditions and accurately defining their functional subsets will facilitate the precise selection of TRMs populations to improve therapeutic efficacy.

What's more, it may hold significant clinical application value to utilise the distribution of TRMs functional subsets as biomarkers for stratifying patients undergoing ICB or TIL therapy to predict clinical outcomes, as well as to adjust the proportions of TRMs populations to enhance therapeutic efficacy.

## 3 Conclusion

TRMs are characterised by being “resident” in peripheral non‐lymphoid tissues (non‐circulating), which can directly kill tumour cells or indirectly exert their cytotoxic potential by recruiting other cells. In‐depth understanding of the spatiotemporal heterogeneity of TRMs and precise definition of their functional subpopulations has two implications for solid tumours: Guiding clinical prognosis; optimising target design to improve the efficacy and safety of ICB and TIL immunotherapy. Therefore, how to characterise TRM subpopulations more comprehensively and in detail through biomarkers to achieve precise immunotherapy of solid tumours is worthy of our continuous exploration.

## Author Contributions

All of the authors had participated in the preparation of this article. Concept and design: Weiqin Jiang, Yinjun He, Xiang Zhang, Guosheng Wu, Weixiang Zhong, and Yile Shang. Manuscript writing and editing: Yile Shang, Weiqin Jiang, Wenguang He, and Xile Zhou. Manuscript revising: Yile Shang, Weiqin Jiang, Hanju Hua, and Feng Ye. Figures 1, 2, 3, 4 preparation: Yile Shang, Yandong Li, and Weiqin Jiang. All authors reviewed the manuscript.

## Ethics Statement

The authors have nothing to report.

## Conflicts of Interest

The authors declare no conflicts of interest.

## Data Availability

The authors have nothing to report.

## References

[1] T. Gebhardt , L. M. Wakim , L. Eidsmo , P. C. Reading , W. R. Heath , and F. R. Carbone , “Memory T Cells in Nonlymphoid Tissue That Provide Enhanced Local Immunity During Infection With Herpes Simplex Virus,” Nature Immunology 10 (2009): 524–530. 19305395 10.1038/ni.1718

[2] X. Jiang , R. A. Clark , L. Liu , A. J. Wagers , R. C. Fuhlbrigge , and T. S. Kupper , “Skin Infection Generates Non‐migratory Memory CD8+ TRM Cells Providing Global Skin Immunity,” Nature 483 (2012): 227–231. 22388819 10.1038/nature10851 PMC3437663

[3] J. M. Schenkel , K. A. Fraser , L. K. Beura , K. E. Pauken , V. Vezys , and D. Masopust , “Resident Memory CD8 T Cells Trigger Protective Innate and Adaptive Immune Responses,” Science 346 (2014): 98–101. 25170049 10.1126/science.1254536 PMC4449618

[4] R. A. Clark , R. Watanabe , J. E. Teague , et al., “Skin Effector Memory T Cells Do Not Recirculate and Provide Immune Protection in Alemtuzumab‐Treated CTCL Patients,” Science Translational Medicine 4 (2012): 117ra7. 10.1126/scitranslmed.3003008 PMC3373186 22261031

[5] K. Okła , D. L. Farber , and W. Zou , “Tissue‐Resident Memory T Cells in Tumor Immunity and Immunotherapy,” Journal of Experimental Medicine 218 (2021): e20201605. 33755718 10.1084/jem.20201605 PMC7992502

[6] J. M. Schenkel , K. A. Fraser , V. Vezys , and D. Masopust , “Sensing and Alarm Function of Resident Memory CD8+ T Cells,” Nature Immunology 14 (2013): 509–513. 23542740 10.1038/ni.2568 PMC3631432

[7] B. Lin , L. Du , H. Li , et al., “Tumor‐Infiltrating Lymphocytes: Warriors Fight Against Tumors Powerfully,” Biomedicine & Pharmacotherapy 132 (2020): 110–873. 10.1016/j.biopha.2020.110873 33068926

[8] M. Buggert , D. A. Price , L. K. Mackay , and M. R. Betts , “Human Circulating and Tissue‐Resident Memory CD8+ T Cells,” Nature Immunology 24 (2023): 1076–1086. 37349380 10.1038/s41590-023-01538-6

[9] P. A. Szabo , M. Miron , and D. L. Farber , “Location, Location, Location: Tissue Resident Memory T Cells in Mice and Humans,” Science Immunology 4 (2019): eaas9673. 30952804 10.1126/sciimmunol.aas9673 PMC6778482

[10] L. K. Mackay , A. Braun , B. L. Macleod , et al., “Cutting Edge: CD69 Interference With Sphingosine‐1‐Phosphate Receptor Function Regulates Peripheral T Cell Retention,” Journal of Immunology 194 (2015): 2059–2063. 10.4049/jimmunol.1402256 25624457

[11] A. Baeyens , V. Fang , C. Chen , and S. R. Schwab , “Exit Strategies: S1P Signaling and T Cell Migration,” Trends in Immunology 36 (2015): 778–787. 26596799 10.1016/j.it.2015.10.005 PMC4832571

[12] C. N. Skon , J. Y. Lee , K. G. Anderson , D. Masopust , K. A. Hogquist , and S. C. Jameson , “Transcriptional Downregulation of S1pr1 Is Required for the Establishment of Resident Memory CD8+ T Cells,” Nature Immunology 14 (2013): 1285–1293. 24162775 10.1038/ni.2745 PMC3844557

[13] F. Mami‐Chouaib , C. Blanc , S. Corgnac , et al., “Resident Memory T Cells, Critical Components in Tumor Immunology,” Journal for Immunotherapy of Cancer 6 (2018): 87. 30180905 10.1186/s40425-018-0399-6 PMC6122734

[14] D. Amsen , K. P. J. M. van Gisbergen , P. Hombrink , and R. A. W. van Lier , “Tissue‐Resident Memory T Cells at the Center of Immunity to Solid Tumors,” Nature Immunology 19 (2018): 538–546. 29777219 10.1038/s41590-018-0114-2

[15] Š. Konjar , X. Ficht , M. Iannacone , and M. Veldhoen , “Heterogeneity of Tissue Resident Memory T Cells,” Immunology Letters 245 (2022): 1–7. 35346744 10.1016/j.imlet.2022.02.009

[16] L. K. Mackay , A. Rahimpour , J. Z. Ma , et al., “The Developmental Pathway for CD103 + CD8+ Tissue‐Resident Memory T Cells of Skin,” Nature Immunology 14 (2013): 1294–1301. 24162776 10.1038/ni.2744

[17] K. Chen , X. Gu , S. Yang , et al., “Research Progress on Intestinal Tissue‐Resident Memory T Cells in Inflammatory Bowel Disease,” Scandinavian Journal of Immunology 98 (2023): e13332. 38441381 10.1111/sji.13332

[18] D. Herndler‐Brandstetter , H. Ishigame , R. Shinnakasu , et al., “KLRG1(+) Effector CD8(+) T Cells Lose KLRG1, Differentiate Into all Memory T Cell Lineages, and Convey Enhanced Protective Immunity,” Immunity 48 (2018): 716–729.e718. 29625895 10.1016/j.immuni.2018.03.015 PMC6465538

[19] R. Fonseca , L. K. Beura , C. F. Quarnstrom , et al., “Developmental Plasticity Allows Outside‐In Immune Responses by Resident Memory T Cells,” Nature Immunology 21 (2020): 412–421. 32066954 10.1038/s41590-020-0607-7 PMC7096285

[20] M. M. Klicznik , P. A. Morawski , B. Höllbacher , et al., “Human CD4 + CD103+ Cutaneous Resident Memory T Cells Are Found in the Circulation of Healthy Individuals,” Science Immunology 4 (2019): eaav8995. 31278120 10.1126/sciimmunol.aav8995 PMC7057121

[21] J. Strobl , L. M. Gail , L. Kleissl , et al., “Human Resident Memory T Cells Exit the Skin and Mediate Systemic Th2‐Driven Inflammation,” Journal of Experimental Medicine 218 (2021): e20210417. 34643646 10.1084/jem.20210417 PMC8563284

[22] B. Rodger , A. J. Stagg , and J. O. Lindsay , “The Role of Circulating T Cells With a Tissue Resident Phenotype (Ex‐T(RM)) in Health and Disease,” Frontiers in Immunology 15 (2024): 1415914. 38817613 10.3389/fimmu.2024.1415914 PMC11137204

[23] J. W. Dean , E. Y. Helm , Z. Fu , et al., “The Aryl Hydrocarbon Receptor Cell Intrinsically Promotes Resident Memory CD8(+) T Cell Differentiation and Function,” Cell Reports 42 (2023): 111963. 36640340 10.1016/j.celrep.2022.111963 PMC9940759

[24] L. K. Mackay , M. Minnich , N. A. M. Kragten , et al., “Hobit and Blimp1 Instruct a Universal Transcriptional Program of Tissue Residency in Lymphocytes,” Science 352 (2016): 459–463. 27102484 10.1126/science.aad2035

[25] Z. Qiu , T. H. Chu , and B. S. Sheridan , “TGF‐β: Many Paths to CD103+ CD8 T Cell Residency,” Cells 10 (2021): 989. 33922441 10.3390/cells10050989 PMC8145941

[26] N. S. Kurd , Z. He , T. L. Louis , et al., “Early Precursors and Molecular Determinants of Tissue‐Resident Memory CD8(+) T Lymphocytes Revealed by Single‐Cell RNA Sequencing,” Science Immunology 5 (2020): eaaz6894. 32414833 10.1126/sciimmunol.aaz6894 PMC7341730

[27] C. M. Carlson , B. T. Endrizzi , J. Wu , et al., “Kruppel‐Like Factor 2 Regulates Thymocyte and T‐Cell Migration,” Nature 442 (2006): 299–302. 16855590 10.1038/nature04882

[28] M. Julve , M. P. Lythgoe , J. Larkin , and A. J. S. Furness , “Lifileucel: The First Cellular Therapy Approved for Solid Tumours,” Trends Cancer 10 (2024): 475–477. 38724322 10.1016/j.trecan.2024.04.003

[29] S. T. Paijens , A. Vledder , M. de Bruyn , and H. W. Nijman , “Tumor‐Infiltrating Lymphocytes in the Immunotherapy Era,” Cellular & Molecular Immunology 18 (2020): 842–859. 33139907 10.1038/s41423-020-00565-9 PMC8115290

[30] A. P. Ganesan , J. Clarke , O. Wood , et al., “Tissue‐Resident Memory Features Are Linked to the Magnitude of Cytotoxic T Cell Responses in Human Lung Cancer,” Nature Immunology 18 (2017): 940–950. 28628092 10.1038/ni.3775 PMC6036910

[31] M. E. Dudley , J. R. Wunderlich , T. E. Shelton , J. Even , and S. A. Rosenberg , “Generation of Tumor‐Infiltrating Lymphocyte Cultures for Use in Adoptive Transfer Therapy for Melanoma Patients,” Journal of Immunotherapy 26 (2003): 332–342. 12843795 10.1097/00002371-200307000-00005 PMC2305721

[32] S. E. Stanton , S. Adams , and M. L. Disis , “Variation in the Incidence and Magnitude of Tumor‐Infiltrating Lymphocytes in Breast Cancer Subtypes,” JAMA Oncology 2 (2016): 1354–1360. 27355489 10.1001/jamaoncol.2016.1061

[33] A. A. Sarnaik , P. Hwu , J. J. Mulé , and S. Pilon‐Thomas , “Tumor‐Infiltrating Lymphocytes: A New Hope,” Cancer Cell 42 (2024): 1315–1318. 39029463 10.1016/j.ccell.2024.06.015

[34] V. Leko and S. A. Rosenberg , “Identifying and Targeting Human Tumor Antigens for T Cell‐Based Immunotherapy of Solid Tumors,” Cancer Cell 38 (2020): 454–472. 32822573 10.1016/j.ccell.2020.07.013 PMC7737225

[35] P. Savas , B. Virassamy , C. Ye , et al., “Single‐Cell Profiling of Breast Cancer T Cells Reveals a Tissue‐Resident Memory Subset Associated With Improved Prognosis,” Nature Medicine 24, no. 7 (2018): 986–993, 10.1038/s41591-018-0078-7. 29942092

[36] Y. Shen , X.‐l. Li , Y.‐x. Li , et al., “Distribution, Phenotype, Functional and Clinical Relevance of CD8 + CD103+ Tissue‐Resident Memory T Cells in Human Gastric Cancer,” Cancer Immunology, Immunotherapy 71, no. 7 (2021): 1645–1654, 10.1007/s00262-021-03105-0. 34767045 PMC10992218

[37] A. Betof Warner , P. G. Corrie , and O. Hamid , “Tumor‐Infiltrating Lymphocyte Therapy in Melanoma: Facts to the Future,” Clinical Cancer Research 29 (2023): 1835–1854. 36485001 10.1158/1078-0432.CCR-22-1922 PMC10183807

[38] Y. Zhang , P. Naderi Yeganeh , H. Zhang , et al., “Tumor Editing Suppresses Innate and Adaptive Antitumor Immunity and Is Reversed by Inhibiting DNA Methylation,” Nature Immunology 25 (2024): 1858–1870. 39169233 10.1038/s41590-024-01932-8

[39] N. R. Maimela , S. Liu , and Y. Zhang , “Fates of CD8+ T Cells in Tumor Microenvironment,” Computational and Structural Biotechnology Journal 17 (2019): 1–13. 30581539 10.1016/j.csbj.2018.11.004 PMC6297055

[40] C. M. Anadon , X. Yu , K. Hänggi , et al., “Ovarian Cancer Immunogenicity Is Governed by a Narrow Subset of Progenitor Tissue‐Resident Memory T Cells,” Cancer Cell 40 (2022): 545–557.e513. 35427494 10.1016/j.ccell.2022.03.008 PMC9096229

[41] M. Philip and A. Schietinger , “CD8(+) T Cell Differentiation and Dysfunction in Cancer,” Nature Reviews. Immunology 22 (2022): 209–223. 10.1038/s41577-021-00574-3 PMC9792152 34253904

[42] X. Song , G. Zhao , G. Wang , and H. Gao , “Heterogeneity and Differentiation Trajectories of Infiltrating CD8+ T Cells in Lung Adenocarcinoma,” Cancers 14 (2022): 5183. 36358600 10.3390/cancers14215183 PMC9658355

[43] M. Sade‐Feldman , K. Yizhak , S. L. Bjorgaard , et al., “Defining T Cell States Associated With Response to Checkpoint Immunotherapy in Melanoma,” Cell 175 (2018): 998–1013.e1020. 30388456 10.1016/j.cell.2018.10.038 PMC6641984

[44] B. Virassamy , F. Caramia , P. Savas , et al., “Intratumoral CD8+ T Cells With a Tissue‐Resident Memory Phenotype Mediate Local Immunity and Immune Checkpoint Responses in Breast Cancer,” Cancer Cell 41 (2023): 585–601.e588. 36827978 10.1016/j.ccell.2023.01.004

[45] S. Corgnac , I. Malenica , L. Mezquita , et al., “CD103+ CD8+ TRM Cells Accumulate in Tumors of Anti‐PD‐1‐Responder Lung Cancer Patients and Are Tumor‐Reactive Lymphocytes Enriched With Tc17,” Cell Reports Medicine 1 (2020): 100–127. 10.1016/j.xcrm.2020.100127 PMC7659589 33205076

[46] J. Deng , A. Thennavan , S. Shah , et al., “Serial Single‐Cell Profiling Analysis of Metastatic TNBC During nab‐Paclitaxel and Pembrolizumab Treatment,” Breast Cancer Research and Treatment 185, no. 1 (2020): 85–94, 10.1007/s10549-020-05936-4. 32949350 PMC8170702

[47] J.‐M. Chauvin and H. M. Zarour , “TIGIT in Cancer Immunotherapy,” Journal for Immunotherapy of Cancer 8 (2020): e000957. 32900861 10.1136/jitc-2020-000957 PMC7477968

[48] J. Yeo , M. Ko , D. H. Lee , Y. Park , and H. S. Jin , “TIGIT/CD226 Axis Regulates Anti‐Tumor Immunity,” Pharmaceuticals (Basel) 14 (2021): 200. 33670993 10.3390/ph14030200 PMC7997242

[49] F. Jiang , M. Mao , S. Jiang , et al., “PD‐1 and TIGIT Coexpressing CD8 + CD103 + Tissue‐Resident Memory Cells in Endometrial Cancer as Potential Targets for Immunotherapy,” International Immunopharmacology 127 (2024): 111–381. 10.1016/j.intimp.2023.111381 38150880

[50] H. Pearce , W. Croft , S. M. Nicol , et al., “Tissue‐Resident Memory T Cells in Pancreatic Ductal Adenocarcinoma Coexpress PD‐1 and TIGIT and Functional Inhibition Is Reversible by Dual Antibody Blockade,” Cancer Immunology Research 11 (2023): 435–449. 36689623 10.1158/2326-6066.CIR-22-0121 PMC10068448

[51] H. S. Han , S. Jeong , H. Kim , et al., “TOX‐Expressing Terminally Exhausted Tumor‐Infiltrating CD8(+) T Cells Are Reinvigorated by Co‐Blockade of PD‐1 and TIGIT in Bladder Cancer,” Cancer Letters 499 (2021): 137–147. 33249194 10.1016/j.canlet.2020.11.035

[52] W. A. Freed‐Pastor , L. J. Lambert , Z. A. Ely , et al., “The CD155/TIGIT Axis Promotes and Maintains Immune Evasion in Neoantigen‐Expressing Pancreatic Cancer,” Cancer Cell 39 (2021): 1342–1360.e1314. 34358448 10.1016/j.ccell.2021.07.007 PMC8511341

[53] K. L. Banta , X. Xu , A. S. Chitre , et al., “Mechanistic Convergence of the TIGIT and PD‐1 Inhibitory Pathways Necessitates Co‐Blockade to Optimize Anti‐Tumor CD8(+) T Cell Responses,” Immunity 55 (2022): 512–526.e519. 35263569 10.1016/j.immuni.2022.02.005 PMC9287124

[54] F. G. Herrera , C. Ronet , M. Ochoa de Olza , et al., “Low‐Dose Radiotherapy Reverses Tumor Immune Desertification and Resistance to Immunotherapy,” Cancer Discovery 12 (2022): 108–133. 34479871 10.1158/2159-8290.CD-21-0003 PMC9401506

[55] S. Demaria , C. N. Coleman , and S. C. Formenti , “Radiotherapy: Changing the Game in Immunotherapy,” Trends Cancer 2 (2016): 286–294. 27774519 10.1016/j.trecan.2016.05.002 PMC5070800

[56] M. Jarosz‐Biej , R. Smolarczyk , T. Cichon , and N. Kulach , “Tumor Microenvironment as A “Game Changer” in Cancer Radiotherapy,” International Journal of Molecular Sciences 20 (2019): 3212. 31261963 10.3390/ijms20133212 PMC6650939

[57] Z. Y. Lin , P. Zhang , P. Chi , et al., “Neoadjuvant Short‐Course Radiotherapy Followed by Camrelizumab and Chemotherapy in Locally Advanced Rectal Cancer (UNION): Early Outcomes of a Multicenter Randomized Phase III Trial,” Annals of Oncology 35 (2024): 882–891. 38964714 10.1016/j.annonc.2024.06.015

[58] W. W. Xiao , G. Chen , Y. H. Gao , et al., “Effect of Neoadjuvant Chemoradiotherapy With or Without PD‐1 Antibody Sintilimab in pMMR Locally Advanced Rectal Cancer: A Randomized Clinical Trial,” Cancer Cell 42 (2024): 1570–1581.e4. 39094560 10.1016/j.ccell.2024.07.004

[59] F. Wang , S. Yue , Q. Huang , et al., “Cellular Heterogeneity and Key Subsets of Tissue‐Resident Memory T Cells in Cervical Cancer,” NPJ Precision Oncology 8 (2024): 145. 39014148 10.1038/s41698-024-00637-3 PMC11252146

[60] A. Arina , M. Beckett , C. Fernandez , et al., “Tumor‐Reprogrammed Resident T Cells Resist Radiation to Control Tumors,” Nature Communications 10 (2019): 3959. 10.1038/s41467-019-11906-2 PMC6718618 31477729

[61] P. Bajpai , S. Agarwal , F. Afaq , et al., “Combination of Dual JAK/HDAC Inhibitor With Regorafenib Synergistically Reduces Tumor Growth, Metastasis, and Regorafenib‐Induced Toxicity in Colorectal Cancer,” Journal of Experimental & Clinical Cancer Research 43 (2024): 192. 38992681 10.1186/s13046-024-03106-8 PMC11238352

[62] S. Fang , H. Cao , J. Liu , G. Cao , and T. Li , “Antitumor Effects of IOX1 Combined With Bevacizumab‐Induced Apoptosis and Immunity on Colorectal Cancer Cells,” International Immunopharmacology 141 (2024): 112–896. 10.1016/j.intimp.2024.112896 39146782

[63] L. M. Tseng , K.‐Y. Lau , J.‐L. Chen , et al., “Regorafenib Induces Damage‐Associated Molecular Patterns, Cancer Cell Death and Immune Modulatory Effects in a Murine Triple Negative Breast Cancer Model,” Experimental Cell Research 429, no. 1 (2023): 113652, 10.1016/j.yexcr.2023.113652. 37209991

[64] T. Duhen , R. Duhen , R. Montler , et al., “Co‐Expression of CD39 and CD103 Identifies Tumor‐Reactive CD8 T Cells in Human Solid Tumors,” Nature Communications 9 (2018): 2724. 10.1038/s41467-018-05072-0 PMC6045647 30006565

[65] C. Lai , G. Coltart , A. Shapanis , et al., “CD8 + CD103+ Tissue‐Resident Memory T Cells Convey Reduced Protective Immunity in Cutaneous Squamous Cell Carcinoma,” Journal for Immunotherapy of Cancer 9 (2021): e001807. 33479027 10.1136/jitc-2020-001807 PMC7825273

[66] L. S. Christian , L. Wang , B. Lim , et al., “Resident Memory T Cells in Tumor‐Distant Tissues Fortify Against Metastasis Formation,” Cell Reports 35 (2021): 109–118. 10.1016/j.celrep.2021.109118 PMC8204287 33979626

[67] Y. Pan , T. Tian , C. O. Park , et al., “Survival of Tissue‐Resident Memory T Cells Requires Exogenous Lipid Uptake and Metabolism,” Nature 543 (2017): 252–256. 28219080 10.1038/nature21379 PMC5509051

[68] H. Frizzell , R. Fonseca , S. N. Christo , et al., “Organ‐Specific Isoform Selection of Fatty Acid–Binding Proteins in Tissue‐Resident Lymphocytes,” Science Immunology 5 (2020): eaay9283. 32245887 10.1126/sciimmunol.aay9283

[69] Y. H. Lin , H. G. Duong , A. E. Limary , et al., “Small Intestine and Colon Tissue‐Resident Memory CD8+ T Cells Exhibit Molecular Heterogeneity and Differential Dependence on Eomes,” Immunity 56 (2023): 207–223.e208. 36580919 10.1016/j.immuni.2022.12.007 PMC9904390

[70] M. H. Yakou , S. Ghilas , K. Tran , et al., “TCF‐1 Limits Intraepithelial Lymphocyte Antitumor Immunity in Colorectal Carcinoma,” Science Immunology 8 (2023): eadf2163. 37801516 10.1126/sciimmunol.adf2163

[71] A. L. Doedens , A. T. Phan , M. H. Stradner , et al., “Hypoxia‐Inducible Factors Enhance the Effector Responses of CD8(+) T Cells to Persistent Antigen,” Nature Immunology 14 (2013): 1173–1182. 24076634 10.1038/ni.2714 PMC3977965

[72] J. H. Kim , J. W. Han , Y. J. Choi , et al., “Functions of Human Liver CD69(+)CD103(−)CD8(+) T Cells Depend on HIF‐2alpha Activity in Healthy and Pathologic Livers,” Journal of Hepatology 72 (2020): 1170–1181. 31987989 10.1016/j.jhep.2020.01.010

[73] L. M. Bartsch , M. P. S. Damasio , S. Subudhi , and H. K. Drescher , “Tissue‐Resident Memory T Cells in the Liver‐Unique Characteristics of Local Specialists,” Cells 9 (2020): 2457. 33187162 10.3390/cells9112457 PMC7696520

[74] J. T. Crowl , M. Heeg , A. Ferry , et al., “Tissue‐Resident Memory CD8+ T Cells Possess Unique Transcriptional, Epigenetic and Functional Adaptations to Different Tissue Environments,” Nature Immunology 23 (2022): 1121–1131. 35761084 10.1038/s41590-022-01229-8 PMC10041538

[75] A. Byrne , P. Savas , S. Sant , et al., “Tissue‐Resident Memory T Cells in Breast Cancer Control and Immunotherapy Responses,” Nature Reviews Clinical Oncology 17 (2020): 341–348. 10.1038/s41571-020-0333-y 32112054

[76] S. Ida , H. Takahashi , R. Kawabata‐Iwakawa , et al., “Tissue‐Resident Memory T Cells Correlate With the Inflammatory Tumor Microenvironment and Improved Prognosis in Head and Neck Squamous Cell Carcinoma,” Oral Oncology 122 (2021): 105–508. 10.1016/j.oraloncology.2021.105508 34507204

[77] Y. Xiao , H. Li , L. Mao , et al., “CD103+ T and Dendritic Cells Indicate a Favorable Prognosis in Oral Cancer,” Journal of Dental Research 98 (2019): 1480–1487. 31658426 10.1177/0022034519882618

[78] F. L. Komdeur , T. M. Prins , S. van de Wall , et al., “CD103+ Tumor‐Infiltrating Lymphocytes Are Tumor‐Reactive Intraepithelial CD8+ T Cells Associated With Prognostic Benefit and Therapy Response in Cervical Cancer,” Oncoimmunology 6 (2017): e1338230. 28932636 10.1080/2162402X.2017.1338230 PMC5599086

[79] F. Djenidi , J. Adam , A. Goubar , et al., “CD8 + CD103+ Tumor–Infiltrating Lymphocytes Are Tumor‐Specific Tissue‐Resident Memory T Cells and a Prognostic Factor for Survival in Lung Cancer Patients,” Journal of Immunology 194 (2015): 3475–3486. 10.4049/jimmunol.1402711 25725111

[80] C. E. Weeden , V. Gayevskiy , C. Marceaux , et al., “Early Immune Pressure Initiated by Tissue‐Resident Memory T Cells Sculpts Tumor Evolution in Non‐small Cell Lung Cancer,” Cancer Cell 41 (2023): 837–852.e836. 37086716 10.1016/j.ccell.2023.03.019

[81] J. S. O'Donnell , M. W. L. Teng , and M. J. Smyth , “Cancer Immunoediting and Resistance to T Cell‐Based Immunotherapy,” Nature Reviews Clinical Oncology 16 (2018): 151–167. 10.1038/s41571-018-0142-8 30523282

[82] A. Kalbasi and A. Ribas , “Tumour‐Intrinsic Resistance to Immune Checkpoint Blockade,” Nature Reviews Immunology 20 (2019): 25–39. 10.1038/s41577-019-0218-4 PMC8499690 31570880

[83] G. Morad , B. A. Helmink , P. Sharma , and J. A. Wargo , “Hallmarks of Response, Resistance, and Toxicity to Immune Checkpoint Blockade,” Cell 184 (2021): 5309–5337. 34624224 10.1016/j.cell.2021.09.020 PMC8767569

[84] A. J. Korman , S. C. Garrett‐Thomson , and N. Lonberg , “The Foundations of Immune Checkpoint Blockade and the Ipilimumab Approval Decennial,” Nature Reviews. Drug Discovery 21 (2022): 509–528. 34937915 10.1038/s41573-021-00345-8

[85] Q. Lecocq , M. Keyaerts , N. Devoogdt , and K. Breckpot , “The Next‐Generation Immune Checkpoint LAG‐3 and Its Therapeutic Potential in Oncology: Third Time's a Charm,” International Journal of Molecular Sciences 22 (2020): 75. 33374804 10.3390/ijms22010075 PMC7795594

[86] L. Chocarro , A. Bocanegra , E. Blanco , et al., “Cutting‐Edge: Preclinical and Clinical Development of the First Approved lag‐3 Inhibitor,” Cells 11 (2022): 2351. 35954196 10.3390/cells11152351 PMC9367598

[87] T. W. Kim , P. L. Bedard , P. LoRusso , et al., “Anti‐TIGIT Antibody Tiragolumab Alone or With Atezolizumab in Patients With Advanced Solid Tumors: A Phase 1a/1b Nonrandomized Controlled Trial,” JAMA Oncology 9, no. 11 (2023): 1574–1582, 10.1001/jamaoncol.2023.3867. 37768658 PMC10540058

[88] N. Acharya , C. Sabatos‐Peyton , and A. C. Anderson , “Tim‐3 Finds Its Place in the Cancer Immunotherapy Landscape,” Journal for Immunotherapy of Cancer 8 (2020): e000911. 32601081 10.1136/jitc-2020-000911 PMC7326247

[89] K. O. Dixon , M. Tabaka , M. A. Schramm , et al., “TIM‐3 Restrains Anti‐Tumour Immunity by Regulating Inflammasome Activation,” Nature 595 (2021): 101–106. 34108686 10.1038/s41586-021-03626-9 PMC8627694

[90] J. J. Harding , V. Moreno , Y. J. Bang , et al., “Blocking TIM‐3 in Treatment‐Refractory Advanced Solid Tumors: A Phase Ia/b Study of LY3321367 With or Without an Anti‐PD‐L1 Antibody,” Clinical Cancer Research 27 (2021): 2168–2178. 33514524 10.1158/1078-0432.CCR-20-4405

[91] S. Hong , Q. Yuan , H. Xia , et al., “Analysis of VISTA Expression and Function in Renal Cell Carcinoma Highlights VISTA as a Potential Target for Immunotherapy,” Protein & Cell 10 (2019): 840–845. 31236852 10.1007/s13238-019-0642-z PMC6834738

[92] L. Yuan , J. Tatineni , K. M. Mahoney , and G. J. Freeman , “VISTA: A Mediator of Quiescence and a Promising Target in Cancer Immunotherapy,” Trends in Immunology 42 (2021): 209–227. 33495077 10.1016/j.it.2020.12.008 PMC8088836

[93] L. Borst , S. H. van der Burg , and T. van Hall , “The NKG2A‐HLA‐E Axis as a Novel Checkpoint in the Tumor Microenvironment,” Clinical Cancer Research 26 (2020): 5549–5556. 32409305 10.1158/1078-0432.CCR-19-2095

[94] R. S. Bhatt , A. Berjis , J. C. Konge , et al., “KIR3DL3 Is an Inhibitory Receptor for HHLA2 That Mediates an Alternative Immunoinhibitory Pathway to PD1,” Cancer Immunology Research 9 (2021): 156–169. 33229411 10.1158/2326-6066.CIR-20-0315 PMC8284010

[95] Y. Simoni , E. Becht , M. Fehlings , et al., “Bystander CD8+ T Cells Are Abundant and Phenotypically Distinct in Human Tumour Infiltrates,” Nature 557 (2018): 575–579. 29769722 10.1038/s41586-018-0130-2

[96] F. P. Canale , M. C. Ramello , N. Núñez , et al., “CD39 Expression Defines Cell Exhaustion in Tumor‐Infiltrating CD8+ T Cells,” Cancer Research 78 (2018): 115–128. 29066514 10.1158/0008-5472.CAN-16-2684

[97] S. Talhouni , W. Fadhil , N. P. Mongan , et al., “Activated Tissue Resident Memory T‐Cells (CD8 + CD103 + CD39+) Uniquely Predict Survival in Left Sided “Immune‐Hot” Colorectal Cancers,” Frontiers in Immunology 14 (2023): 1057292, 10.3389/fimmu.2023.1057292. 37251410 PMC10213916

[98] A. Chow , F. Z. Uddin , M. Liu , et al., “The Ectonucleotidase CD39 Identifies Tumor‐Reactive CD8+ T Cells Predictive of Immune Checkpoint Blockade Efficacy in Human Lung Cancer,” Immunity 56 (2023): 93–106.e106. 36574773 10.1016/j.immuni.2022.12.001 PMC9887636

[99] J. J. Milner , C. Toma , B. Yu , et al., “Runx3 Programs CD8+ T Cell Residency in Non‐lymphoid Tissues and Tumours,” Nature 552 (2017): 253–257. 29211713 10.1038/nature24993 PMC5747964

